# Clinical Heterogeneity of Acquired Idiopathic Isolated Adrenocorticotropic Hormone Deficiency

**DOI:** 10.3389/fendo.2021.578802

**Published:** 2021-02-19

**Authors:** Yasunori Fujita, Hironori Bando, Genzo Iguchi, Keiji Iida, Hitoshi Nishizawa, Keitaro Kanie, Kenichi Yoshida, Ryusaku Matsumoto, Kentaro Suda, Hidenori Fukuoka, Wataru Ogawa, Yutaka Takahashi

**Affiliations:** ^1^Division of Diabetes and Endocrinology, Department of Internal Medicine, Kobe University Graduate School of Medicine, Kobe, Japan; ^2^Medical Center for Student Health, Kobe University, Kobe, Japan; ^3^Division of Biosignal Pathophysiology, Kobe University, Kobe, Japan; ^4^Division of Diabetes and Endocrinology, Hyogo Prefectural Kakogawa Medical Center, Kakogawa, Japan; ^5^Department of Diabetes and Endocrinology, Nara Medical University, Kashihara, Japan

**Keywords:** isolated ACTH deficiency, hypopituitarism, anti-pituitary antibody, anti-corticotroph antibody, anti-follicular stellate cell antibody, classification, principal component analyses, cluster analyses

## Abstract

**Objective:**

Heterogeneous clinical characteristics are observed in acquired isolated adrenocorticotropic hormone (ACTH) deficiency (IAD); however, its classification remains to be established because of its largely unknown pathophysiology. In IAD, anti-pituitary antibodies have been detected in some patients, although their significance remains unclear. Therefore, this study aimed to classify patients with IAD and to clarify the significance of anti-pituitary antibodies.

**Design and Methods:**

We analyzed 46 consecutive patients with IAD. Serum anti-pituitary antibodies were analyzed *via* immunofluorescence staining using a mouse pituitary tissue. Principal component and cluster analyses were performed to classify IAD patients based on clinical characteristics and autoantibodies.

**Results:**

Immunofluorescence analysis using the sera revealed that 58% of patients showed anti-corticotroph antibodies and 6% of patients showed anti-follicular stellate cell (FSC) antibodies. Principal component analysis demonstrated that three parameters could explain 70% of the patients. Hierarchical cluster analysis showed three clusters: Groups A and B comprised patients who were positive for anti-corticotroph antibodies, and plasma ACTH levels were extremely low. Groups A and B comprised middle-aged or elderly men and middle-aged women, respectively. Group C comprised patients who were positive for the anti-FSC antibody and elderly men; plasma ACTH levels were relatively high.

**Conclusions:**

Patients with IAD were classified into three groups based on clinical characteristics and autoantibodies. The presence of anti-corticotroph antibody suggested severe injury to corticotrophs. This new classification clearly demonstrated the heterogeneity in the pathogenesis of IAD.

## Introduction

Isolated adrenocorticotropic hormone (ACTH) deficiency (IAD) is characterized by secondary adrenal insufficiency with low or no cortisol production and normal secretion of pituitary hormones other than ACTH (1). IAD is categorized as congenital [e.g., T-box transcription factor (*TPIT*) or proopiomelanocortin (*POMC*) mutation (2, 3)] and acquired. The prevalence of acquired IAD was reported to be 3.8 to 7.3 per 100,000 people (4), and the pathogenesis of acquired IAD is mostly unclear. Some cases of acquired IAD have been reportedly associated with autoimmune diseases, including autoimmune thyroid diseases (5); anti-pituitary antibodies, such as anti-corticotroph antibody, in the serum (6, 7); and hypophysitis related with anti-programmed death 1 or anti-programmed death ligand 1 antibodies (8). These data strongly suggest that autoimmune etiology is involved in the pathogenesis of acquired IAD (1, 9). Interestingly, a case of acquired IAD as a form of paraneoplastic syndrome caused by autoimmunity to corticotrophs has been reported (10). However, the pathophysiological significance of these autoantibodies remains unclear.

Several reports have investigated the histopathology of pituitary tissues in acquired IAD cases. In autopsy cases of acquired IAD, the anterior pituitary gland was atrophic with the disappearance of ACTH-positive cells accompanied by fibrosis and infiltration of lymphocytes (11, 12), suggesting that the acquired IAD development was associated with cell-mediated cytotoxicity. Conversely, other autopsy cases showed normal pituitary glands without evidence of inflammation and fibrosis (13). These contrasting reports suggest that the pathogenesis of acquired IAD is heterogeneous; however, its classification remains to be established.

Therefore, this study aimed to classify patients with IAD and to clarify the significance of anti-pituitary antibodies.

## Methods

### Subjects

This study was approved by the ethics committee of the Kobe University Graduate School of Medicine and Hyogo Prefectural Kakogawa Medical Center. Patients provided written informed consent for the analysis. A total of 46 consecutive patients with acquired idiopathic IAD at Kobe University Hospital and Hyogo Prefectural Kakogawa Medical Center between 1992 and 2018 were recruited and analyzed retrospectively (**Table 1**). Acquired IAD was diagnosed as previously described (1). Briefly, to evaluate the corticotroph function, an insulin tolerance test (ITT) was performed; however, a corticotropin-releasing hormone test was performed if ITT was not applicable. In this study, acquired IAD was diagnosed when the hypothalamic-pituitary-adrenal axis was solely impaired (1). Autoimmune thyroid disease was diagnosed with a presence of anti-thyroid antibodies and/or clinical course and the results of thyroid ultra sonography. Our cohort did not include patients with congenital IAD in terms of their clinical course and age at diagnosis. Cases with exogenous steroid administration were carefully excluded.

**Table 1:** Clinical characteristics of patients.

Number of the patients (men/women)		46 (29 (63%)/17 (37%)) (62.8 ± 9.3/50.1 ± 13.0)
Age at diagnosis (men/women)		58.1 ± 12.3 (62.8 ± 9.3/50.1 ± 13.0)
Length of disease duration before blood sampling for anti-pituitary antibodies (years)		7.1 ± 6.0
Plasma ACTH level at diagnosis (men/women) (pg/ml)		5.5 ± 8.9/3.9 ± 5.4
Complicating autoimmune diseases and/or autoantibodies (%)	Autoimmune thyroid diseases*	30
	Other autoimmune diseases**	17
Number of presence of anti-corticotroph antibody		19
Number of presence of anti-FSC antibody		2
Total number (patients)		46

*In fact, fourteen patients (30%) were diagnosed with autoimmune thyroid disease, but eleven patients show anti-thyroid antibodies. Eight chronic thyroiditis, one Graves’ diseases, one patient with anti-Tg, TPO, and TSH receptor antibodies, and one patient with anti-TG and TSH receptor antibodies.

**One patient with rheumatoid arthritis and rheumatic fever, one ulcerative colitis, one immunoglobulin A (IgA) nephropathy, one alopecia areata, and five patients with anti-nuclear antibody.

### Immunofluorescence Staining Using a Mouse Pituitary Tissue

We analyzed the sera obtained from 33 patients (among the 46 patients). Sera from five healthy subjects were used as controls. Mouse experiments were performed according to the guidelines of the Animal Ethics Committee of Kobe University Graduate School of Medicine, and the experimental protocols were approved by the Institutional Animal Care and Use Committee. The detection for anti-pituitary antibodies was based on indirect immunofluorescence methods. The pituitary tissues of 3-month-old C57BL/6 mice were used for immunofluorescence staining. Tissue specimens were fixed in 4% paraformaldehyde, dehydrated in graded ethanol, and embedded in paraffin. For immunofluorescence staining, blocking of Fc receptor and quenching lipofuscin autofluorescence were performed using an Fc receptor blocker (Innovex, Richmond, CA) and True-Black quencher (1:20 in 70% ethanol, Biotium, Hayward, CA), respectively (14). Patients’ serum (25-fold dilution), anti-ACTH antibody (200-fold dilution, Ab74976, Abcam, Cambridge, MA), and anti-S100β antibody (100-fold dilution, Ab52642, Abcam) were used as primary antibodies. Goat anti-human immunoglobulin G Alexa Fluor 488 and donkey anti-rabbit Alexa Fluor 546 were used as secondary antibodies for immunofluorescent signal detection (500-fold dilution, Molecular Probes, Eugene, OR). Antibodies were diluted in Can Get Signal Immunostain B (TOYOBO, Osaka, Japan). Images were obtained using a BZ-8100 microscope (Keyence, Osaka, Japan).

### Statistical Analyses

Statistical analyses were performed using JMP Statistical Database Software version 14.2.0 (Statistical Analysis System Institute, Cary, NC, USA). Principal component analysis (PCA) and hierarchical cluster analyses were performed using six clinical parameters (age at diagnosis, sex, plasma ACTH level at diagnosis, concomitant autoimmune diseases, and positivity for anti-corticotroph antibody or anti-FSC antibody).

## Results

### Clinical Characteristics of Patients

A total of 46 patients with acquired IAD were recruited and analyzed retrospectively (**Table 1**). Patients predominantly comprised men (63%). The age of disease diagnosis was 58.1 ± 12.3 years and was older in men than that in women (62.8 ± 9.3 vs. 50.1 ± 13.0 years). It has been reported that the sex ratio of acquired IAD is slightly predominant in men, and the age at diagnosis of men was older than that of women (15). Our cohort was consistent with this report. Moreover, 28% of patients had complicated autoimmune diseases (eight chronic thyroiditis, one Graves’ disease, one rheumatoid arthritis and rheumatic fever, one ulcerative colitis, one immunoglobulin A nephropathy, and one alopecia areata). These patients were divided into two groups according to the type of complicating autoimmune diseases: autoimmune thyroid diseases and others (**Table 1**). Furthermore, 41% of patients exhibited autoantibodies (11 patients, anti-thyroid antibodies; five, anti-nuclear antibodies; two, rheumatoid factor; and one, single-stranded DNA antibodies) in the serum.

### Anti-Pituitary Autoantibodies

**Figure 1:**
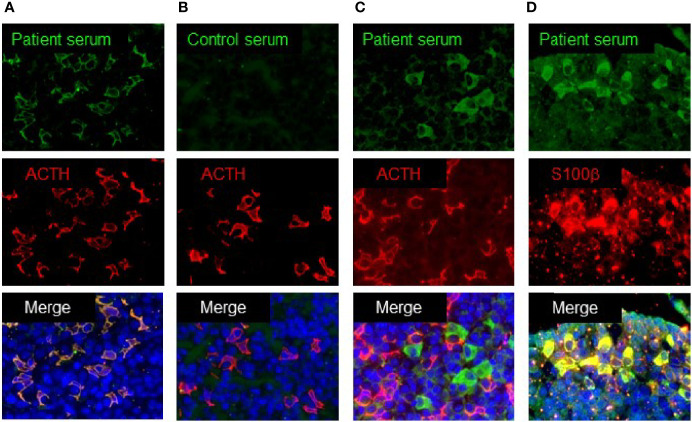
Immunofluorescence analysis using mouse pituitary tissue. **(A)** Patient’s serum recognized ACTH-positive cells. **(B)** Control serum showed negative staining. **(C)** Patient’s serum recognized ACTH-negative cells. **(D)** Patient’s serum recognized S100b-positive cells.

Some patients with acquired IAD have been reported to exhibit serum anti-corticotroph antibodies (16). We analyzed the serum anti-pituitary antibodies in 33 patients using the mouse pituitary tissue by immunofluorescence staining. Among them, 19 (58%) showed the presence of anti-corticotroph antibody in the sera. **Figure 1A** shows the positive staining of pituitary cells, which merged with ACTH-positive cells, indicating the presence of anti-corticotroph antibody. In contrast, 42% of patients showed negative staining (**Figure 1B**). Interestingly, two patients showed positive staining for pituitary cells, but the staining did not merge with ACTH-positive cells (**Figure 1C**). We found that these positive cells merged with S100 β-positive cells, a marker for FSCs (**Figure 1D**). These data clearly demonstrated that autoimmunity to the pituitary in patients with acquired IAD was heterogeneous.

### Classification of Patients Using Principal Component and Cluster Analyses

**Figure 2:**
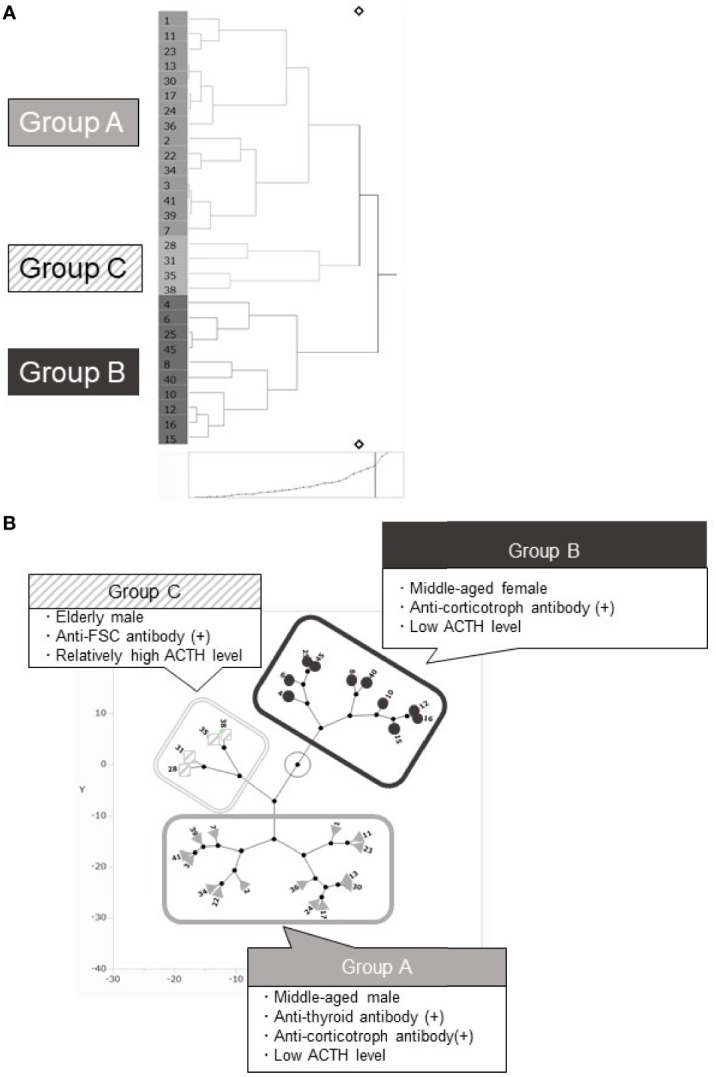
Hierarchical cluster and principal component analysis. **(A)** Hierarchical cluster analysis classified patients into 3 groups: Groups A, B, and C. **(B)** The constellation plots based on the principal component analysis. Three components were clearly discriminated into 3 groups, which were comparable with groups categorized using cluster analysis.

**Figure 3:**
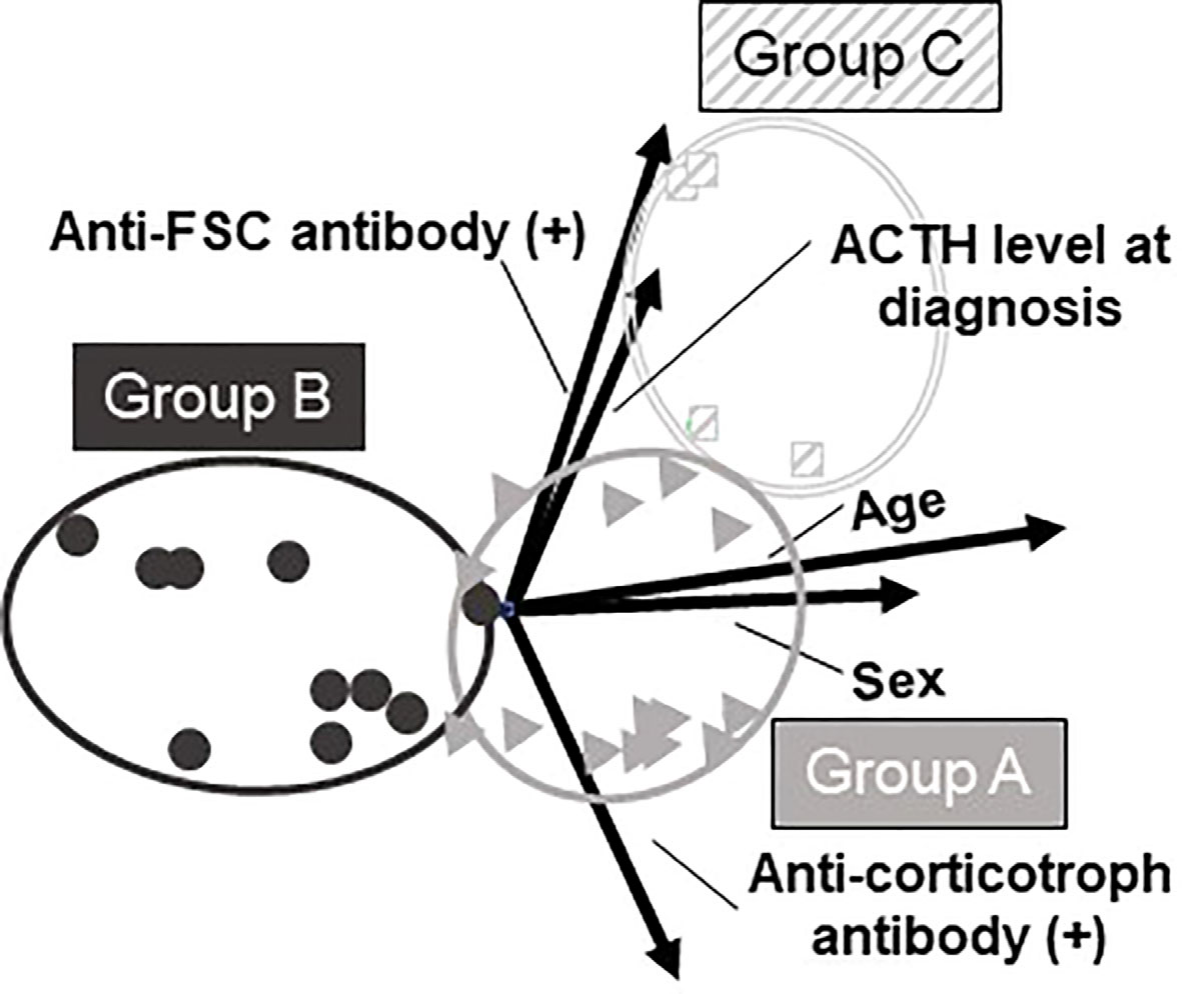
Three-dimensional scatter plot based on PCA and cluster analysis. Three-dimensional scatter plot shows clear discrimination among the 3 groups.

In addition to the patients’ clinical characteristics, we included the information of autoantibodies against the pituitary cells. All the requisite data were available in 29 patients and we analyzed these patients. Patients were classified using PCA and hierarchical cluster analysis. For these analyses, six clinical parameters were included: age at diagnosis, sex, plasma ACTH level at diagnosis, complicating autoimmune diseases, presence of anti-corticotroph antibody, and presence of anti-FSC antibody (**Table 3**). PCA could reduce these six clinical parameters into three new parameters, known as the principal components (PCs). These three PCs could explain approximately 70% (cumulative contribution ratio) of all the data (**Table 2**). When varimax rotation was used, each PC was more clearly described based on parameters showing high correlation: PC 1, age and sex; PC 2, presence of anti-FSC antibody or anti-corticotroph antibody and plasma ACTH level at diagnosis; and PC 3, complicating autoimmune diseases (**Table 3**). Next, hierarchical cluster analysis was performed using the same parameters. This analysis also revealed three clusters: groups A, B, and C (**Figure 2A**) and resulted in the same group classification (**Figure 2B**). The characteristics of these groups are shown in **Table 4**. Groups A and B comprised patients who were positive for the anti-corticotroph antibody with extremely low plasma ACTH levels. Groups A and B comprised middle-aged or elderly men and middle-aged women, respectively. Group C comprised patients who were positive for the anti-FSC antibody and elderly men with relatively high plasma ACTH levels. A three-dimensional scatter plot showed clear discrimination of each cluster of the group, suggesting that these groups had different pathophysiologies (**Figure 3**).

**Table 2:** Principle component analysis using six clinical parameters.

Principal components	Eigenvalue	Contribution ratio	Cummulative
			contribution ratio
1	1.76	29.32	29.32
2	1.38	23.03	52.36
3	1.03	17.18	69.54
4	0.76	12.70	82.24
5	0.63	10.53	92.76
6	0.43	7.24	100.00

**Table 3:** Factor burden for each clinical factor in three principal components.

	PC 1	PC 2	PC 3
Age	**0.86**	0.13	−0.12
Sex	**0.83**	0.02	0.05
ACTH level at diagnosis	0.22	**0.62**	0.25
Complicating autoimmune diseases	−0.07	0.04	**0.97**
Presence of anti-corticotroph antibody	0.28	**−0.78**	0.17
Presence of anti-FSC antibody	0.20	**0.72**	0.02

Clinical factors with a large absolute value of factor burden were shown in bold. It means that these clinical factors are strongly involved in the PC.

**Table 4:** Clinical characteristics of each group.

	Age(years)	Men/Women% (number)	ACTH level at diagnosis (pg/mL)	Autoimmune disease		Anti-corticotroph antibody % (number)	Anti-FSC antibody % (number)	Features
				Thyroid disease % (number)	Other than thyroid disease % (number)			
**Group A** **(N = 15, 52%)**	60.9 ± 10.3	100/0 (15/0)	2.2 ± 5.6	47 (7/15)	20 (3/15)	73 (11/15)	0 (0/15)	Middle-aged or elderly men
								Anti-thyroid antibody (+)
								Anti-corticotroph antibody (+)
								Low ACTH level
**Group B** **(N = 10, 34%)**	45.3 ± 11.2	0/100 (0/10)	3.8 ± 5.8	30 (3/10)	30 (3/10)	60 (6/10)	0 (0/10)	Middle-aged women
								Anti-corticotroph antibody (+)
								Low ACTH level
**Group C** **(N = 4, 14%)**	72.8 ± 3.8	100/0 (4/0)	21.1 ± 10.3	25 (1/4)	0 (0/4)	25 (1/4)	50 (2/4)	Elderly men
								Anti-FSC antibody (+)
								Relatively high ACTH level

## Discussion

As a pathogenesis, acquired IAD is associated with variable conditions, including lymphocytic hypophysitis (17), traumatic brain injury (18), Sheehan’s syndrome (19), and empty sella syndrome (20) on rare occasions. However, most cases are idiopathic (1). Although several lines of evidence suggest that pituitary autoimmunity is involved in the pathogenesis, clinical characteristics are heterogeneous. In this study, we demonstrated that idiopathic acquired IAD can be classified into three groups, based on PCA and cluster analysis using the clinical characteristics and type of anti-pituitary antibodies. We identified two anti-corticotroph antibody-positive groups and one anti-FSC antibody-positive group. Interestingly, clinical characteristics were evidently different between these groups, suggesting that they have different pathophysiologies. Bensing et al. reported that the sera obtained from 34% of patients with acquired IAD showed immunoreactivity toward the 49-kDa and 36-kDa proteins in human pituitary tissues (21). They demonstrated that the sera of 15, 12, and 6% of patients showed immunoreactivity toward the 49-kDa protein, 36-kDa protein, and both, respectively, indicating the presence of various antigens, although the antigen was not identified. Our study demonstrated the two different anti-pituitary antibodies, anti-corticotroph antibody (58%) and anti-FSC antibody (6%). Although anti-corticotroph antibody has been reported in patients with acquired IAD (6, 7), the presence of anti-FSC antibody was first noted in this study. The pathogenesis is suggested to be different among these conditions.

In this study, patients were classified into three groups: groups A and B comprising patients who were positive for the anti-corticotroph antibody with extremely low plasma ACTH levels. Groups A and B comprised middle-aged or elderly men and middle-aged women, respectively. Group C comprised patients who were positive for anti-FSC antibody and elderly men with relatively high plasma ACTH levels. Interestingly, groups A and B comprised patients with anti-corticotroph antibody with extremely low ACTH levels. These data suggest that corticotrophs were severely injured in the presence of anti-corticotroph antibody, although its causal role or destructive results remain unknown. In contrast, the presence of anti-FSC antibody was associated with relatively high ACTH levels in group C, suggesting an indirect or functional impairment of the corticotroph.

FSCs are characterized by S100β expression and have a star-like appearance (22–24). Several functions of FSCs have been proposed including the following: (1) scavenger activity, (2) intercellular communication, (3) stem cell function, and (4) function as sustentacular cells by providing mechanical support and modulating the function of hormone-secreting cells by producing various growth factors and cytokines, such as the basic fibroblast growth factor, vascular endothelial growth factor, interleukin (IL)-6, and leukemia inhibitory factor (25, 26). Several functional links have been reported between FSCs and corticotrophs. Horiguchi et al. succeeded in separating S100β-positive cells into round-cell (dendritic cell-like) and process-cell types (27). They found that dendritic-cell-like round-type S100β-positive cells specifically express a proton-sensitive receptor, Gpr68, and in acidic condition, *via* Gpr68, these cells and respond by the production of IL-6 to suppress POMC expression as a paracrine manner (28). Additionally, CXCL10 and its receptor, CXCR3 were expressed by dendritic-cell-like round-type S100β-positive cells and corticotroph, respectively. IFN-γ induces the expression of CXCL10 in dendritic-cell-like round-type S100β-positive cells. Up-regulated CXCL10 suppress ACTH production in corticotroph *via* CXCL3. Thus, paracrine communication between dendritic-cell-like round-type S100β-positive cells and corticotrophs through the interaction of CXCL10/CXCR3 has been suggested (29). In addition, CXCL10/CXCL3 signaling inhibits CRF-stimulated ACTH secretion (30). Collectively, it is hypothesized that if the anti-FSC antibody has a stimulating ability to promote IL-6 or CXCL10 secretion from FSCs like TSH receptor antibody does in Graves’ disease, a functional impairment of corticotroph may occur, resulting in acquired IAD. To clarify the underlying mechanisms, further investigations are necessary.

Hierarchical cluster analysis clearly categorized acquired IAD into three groups based on clinical characteristics and the presence of anti-pituitary antibodies. In any groups, the ratio of anti-corticotroph antibody or anti-FSC antibody was not 100%. This may be because of the duration between the onset of the disease and timing of blood sampling (**Table 1**). However, as demonstrated by the cluster analysis and PCA, this group classification explains all patients’ comprehensive characteristics. One reason for the inclusion of anti-pituitary antibody-negative patients into these groups was the sensitivity of the assay for these antibodies. Sera were collected during the clinical course; that is, samples were not necessarily collected at the onset of acquired IAD, suggesting that the titer might decrease during the clinical course.

This study has several limitations. First, as discussed above, the sensitivity for detecting an anti-pituitary antibody may not be sufficient. In this study, a mouse pituitary tissue was used for immunofluorescence; the sensitivity may be improved by using human pituitary tissue (14) and hypothetically, some of the data may not reflect the real reactivity profile of human antigens. Second, the total number of patients was insufficient because of the rarity of acquired IAD. Third, sera from all patients were not available, and the timing of blood sampling was not unified. Nonetheless, cluster analysis and PCA clearly categorized these classifications, indicating their clinical relevance. Fourth, we did not analyze the titer of these autoantibodies among the patients, that might allow more precise evaluation.

In conclusion, patients with acquired IAD were classified into three groups based on clinical characteristics and autoantibodies. The presence of anti-corticotroph antibody suggested severe injury of corticotrophs. This new classification may explain the heterogeneity in the pathogenesis of acquired IAD. Although further investigation is necessary, this classification provides an important clue for understanding and clarifying the pathogenesis of acquired IAD.

## Data Availability Statement

The raw data supporting the conclusions of this article will be made available by the authors, without undue reservation.

## Ethics Statement

The studies involving human participants were reviewed and approved by the ethics committee of the Kobe University Graduate School of Medicine and Hyogo Prefectural Kakogawa Medical Center. The patients/participants provided their written informed consent to participate in this study. The animal study was reviewed and approved by the Institutional Animal Care and Use Committee of Kobe University Graduate School of Medicine.

## Author Contributions

YF, HB, and GI analyzed and interpreted the data and wrote the draft of the article. KI, HN, HF, and YT contributed to the preparation of the patient cohort. KK, KY, RM, KS, and WO contributed to data collection. YT contributed to the study supervision and critical revision of the article for important intellectual content. All authors contributed to the article and approved the submitted version.

## Funding

This work was supported by the Japan Society for the Promotion of Science (KAKENHI, grant 23659477 and 26670459 [YT], 15K09431, and 18K08514 [GI], and 17K16165 [HB]), Foundation for Growth Science (GI), Japan Agency for Medical Research and Development (17bm0804012h0001) (YT), Uehara Memorial Foundation (YT), and Naito Foundation (YT).

## Conflict of Interest

The authors declare that the research was conducted in the absence of any commercial or financial relationships that could be construed as a potential conflict of interest.
